# Compartment syndrome following short saphenous varicose vein surgery: a case report

**DOI:** 10.4076/1757-1626-2-7118

**Published:** 2009-07-29

**Authors:** Yousef Shahin, Niteen Tapuria, Peter Lee Chong

**Affiliations:** Department of vascular surgery, Lincoln County HospitalGreetwell Road, Lincoln, LN2 5QYUK

## Abstract

Compartment syndrome is an infrequent but serious complication which can occur post-operatively. In our case, a patient developed severe left calf pain following short saphenous vein surgery. She underwent emergency fasciotomy and made an excellent recovery. Informed consent for varicose vein surgery should probably include the possibility of developing compartment syndrome.

## Introduction

Long and short saphenous varicose vein surgeries are very common procedures. Complications such as bleeding, wound infection, recurrence, paraesthesiae are common, and major complications like DVT/PE, sensory and motor nerve injury (although less common) are well recognised. However, compartment syndrome is not a recognised complication of varicose vein surgery and is not typically mentioned during consent.

## Case presentation

A 41-year-old white Caucasian female presented to our department with severe ipsilateral calf pain (despite analgesia) and swelling approximately 12 hours following left sapheno-popliteal disconnection with excision of the short saphenous vein for symptomatic varicose veins. At operation, iatrogenic bleeding necessitated a call for more experienced assistance and repair of a venous tear close to the junction using 5/0 prolene.

On examination, the leg was firm and swollen, with severe calf tenderness. The left leg circumference measured 38 cm compared with the right which measured 30 cm. Sensation was intact but hyperaesthetic, and distal pulses were palpable with normal capillary refill. Active and passive ankle movements were impossible due to extreme pain.

A clinical diagnosis of posterior leg compartment syndrome was made and our patient taken to theatre for emergency four compartment fasciotomy. At operation, the muscles of the posterior compartment were bulging out on dividing the containing fascia, thus confirming the diagnosis. The deep posterior muscle compartment was also released. There was no evidence of a haematoma or ongoing bleeding. CK level on admission was 81 U/L and fell post fasciotomy to 40 U/L.

A post-operative colour duplex ultrasound scan of the left leg showed a patent popliteal vein with no evidence of deep vein thrombosis. Post-operatively she made a fully functional recovery and was discharged home after 1 week. She was recently seen in the out-patient clinic and underwent complete closure of her fasciotomy wounds.

## Discussion

Compartment syndrome develops where raised pressure within a confined non-elastic space compromises tissue function. This results in a combination of sensory and motor manifestations. Symptoms and signs include severe pain (usually out of proportion to the actual injury), pain on moving distal joints and paraesthesiae. Distal pulses are quite often present and normal. Absent pulses is a late sign or can be indicative of an associated vascular injury [1]. Prompt diagnosis requires a high index of suspicion and timely decompression is essential to prevent or limit morbidity. The diagnosis of compartment syndrome is a clinical one and measurement of compartment pressure, although possible, is usually impractical in the emergency situation, and finds its greatest use in cases where the diagnosis is in doubt.

Compartment syndrome most commonly develops following trauma and limb re-perfusion after a period of ischaemia, but can also occur with burns and with poor positioning, especially during a long procedure [2]. Compartment syndrome as a post-operative complication of long saphenous vein surgery is only reported in a small German case series of three patients [3]. We found no reference to this complication occurring following surgery of the short saphenous vein. In our case, early clinical diagnosis and prompt intervention resulted in a favourable outcome. However, initial consent for varicose vein surgery did not include the possibility of developing compartment syndrome nor the requirement for fasciotomy.

## Conclusion

Compartment syndrome is an unrecognised but potentially serious complication following varicose vein surgery, especially where the operation has been prolonged due to iatrogenic injury. A high index of suspicion is required in any patient presenting with excruciating pain and/or swelling following surgery on the limb. Reduced movement and sensory changes are useful clinical signs. Pulses are often present and palpable and this is a potential caveat for the inexperienced diagnostician. Late diagnosis could result in loss of function or limb loss. Informed consent for varicose vein surgery should probably include the risk of developing compartment syndrome.

## Abbreviations

CK: creatine kinase
DVT: deep vein thrombosis
PE: pulmonary embolism
U/L: Unit per Litre
